# A patient with unusual features and a 69.5 Mb duplication from a *de novo* extra der (9): A case report

**DOI:** 10.3892/mmr.2015.3436

**Published:** 2015-03-05

**Authors:** YU-CHUN ZHOU, CUI ZHANG, JIN-SHENG ZHAI, TIAN-FU LI, QIU-YUE WU, WEI-WEI LI, NA LI, XIAO-JUN LI, YU-FENG HUANG, YING-XIA CUI, XIN-YI XIA

**Affiliations:** 1 Institute of Laboratory Medicine, Jinling Hospital, Nanjing University School of Medicine, Nanjing 210002, P.R. China; 2 Department of Healthcare, Jinling Hospital, Nanjing University School of Medicine, Nanjing 210002, P.R. China

**Keywords:** *de novo* extra der (9), sensorineural hearing loss, ptosis and strabismus of left eye, mild body asymmetry

## Abstract

Partial trisomy 9 is a common autosomal trisomy, which is characterized by non-specific psychomotor delay, mental retardation and moderately abnormal characteristic facial features. Generally, partial trisomy 9 leads to variable phenotypes depending on the size and position of the duplicated region. However, a precise genotype/phenotype map has not been determined. The present study reports the case of a 3-year-old female with certain typical features of trisomy 9p syndrome, who presented with a number of the distinctive symptoms, as well as sensorineural hearing loss, which has not previously been associated with this trisomy. Karyotype, M-FISH and OaCGH analysis were performed on the patient and her parents. The final karyotype was determined to be 47, XX, +mar.ish der (9) (wcp9+). arr cgh 9pterq21.12 (DOCK8→LOC138225)×3. Cytogenetic results showed a *de novo* extra der (9) with 69.5 Mb duplication. Although the molecular mechanism underlying the hearing loss is unclear, it was proposed that the 9q13→9q21 region may be critical for hearing.

## Introduction

Partial trisomy 9 is the fourth most common autosomal trisomy after trisomies 21, 18 and 13. Since Rethoré *et al* (1) reported the first identified case of partial trisomy 9 as a chromosomal anomaly, >150 cases have been described. In addition to non-specific psychomotor delay and mental retardation, common clinical features, including moderately abnormal characteristic facial features, clinodactyly of the 5th fingers, shortened digits, hypoplastic nails, abnormal dermatoglyphics and hypoplastic brain association with Dandy-Walker malformation are observed (2). Trisomy for 9pter-p21 is hypothesized to be responsible for the majority of these features (3). Intrauterine growth retardation, cleft lip/palate, skeletal anomalies and heart defects are more common with trisomic segments extending through 9q22-9q32 (4–7). In general, partial trisomy 9 leads to variable phenotypes dependent upon the size and position of the duplicated region (8). However, a precise genotype/phenotype map has not yet been proposed. The present study describes the case of a 3-year-old female with a number of the typical features of trisomy 9p syndrome, as well as distinctive features that include sensorineural hearing loss and mild body asymmetry. Cytogenetic results showed the presence of a *de novo* extra der (9) with 69.5 Mb duplication.

## Case report

### Case presentation and analysis

**Figure 1:**
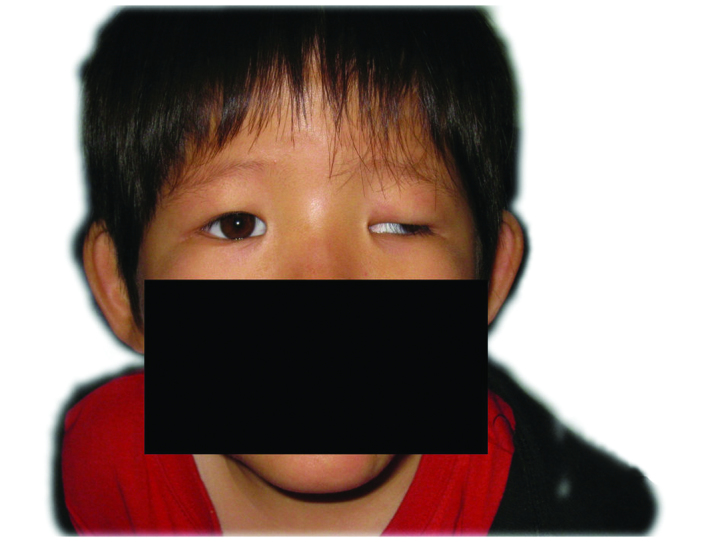
Facial features of a patient with duplication of 9pter→9q21.12.

**Figure 2:**
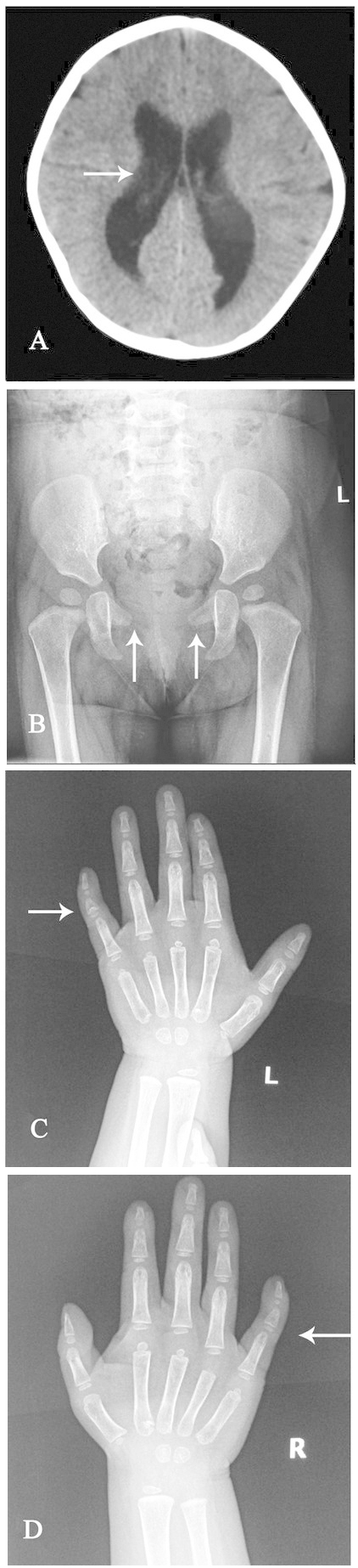
Patient analysis. (A) Cerebral computerized tomography showed enlargement of the lateral ventricles. (B) Roentgenogram of the hypoplastic pubic bones. Roentgenogram of the (C) left hand and (D) right hand, shows the left is smaller than the right and there is clinodactyly of both 5th fingers.

A 3-year-old Chinese female was referred to us for further investigation for mental retardation and hearing loss. The girl was born full-term with uneventful gestation by elective cesarean as the first child of nonconsanguineous parents. The mother and father were 28 and 27 years old, respectively, at her birth. Family history was negative, meaning the other families in this pedigree exhibited no similar ilness. The girl had a birth weight of 3,900 g (95th centile), length of 50 cm (50th centile) and head circumference was 35 cm (50th centile), as well as 1 min and 5 min Apgar scores were of 10, respectively. Her psychomotor development was substantially delayed with severe speech retardation. The patient spoke at the age of 3-years and walked without assistance at the age of 2-years. On examination, the girl had a height of 98.5 cm (77th centile) and weight of 16.5 kg (80th centile). The patient presented with a characteristic face with an antimongoloid slant of palpebral fissures, a broad and prominent nasal bridge, low-set and forwardly-rotated auricles, large poorly lobulated ears and downturned corners of the mouth (Fig. 1). A short neck, clinodactyly of both of the 5th fingers, a bilateral simian crease, joint hyperlaxity and hypoplasia of the toenails were also observed. In addition to the phenotypes of typical trisomy 9p, the patient presented with distinctive features, including the left side of the body slightly smaller than the right with ptosis and strabismus of left eye and sensorineural hearing loss (left ear at 100 decibels, right at 40 decibels). Cerebral computerized tomography showed enlargement of the lateral ventricles (Fig. 2A), 3rd, 4th ventricles and basal cistern, with a mild agenesis of the cerebellar tonsil. Roentgenograms of the skeleton demonstrated hypoplastic pubic bones (Fig. 2B), and bilateral hypoplastic distal phalanges of the feet, pes valgus and bilateral clinodactyly of both 5th fingers (Fig. 2C and D). Cardiac and renal ultrasound findings were normal. This study was approved by the ethics committee of Jinling Hospital, Nanjing University School of Medicine (Nanjing, China), and written informed consent was obtained from the parents.

### Chromosome analysis

#### Karyotype analysis

Karyotyping was performed on peripheral blood lymphocytes from the patient and her parents. Peripheral blood lymphocyte cultures were cultivated using RPMI media supplemented with 10% fetal calf serum (Lai Fu institute of biotechnology, Qing Dao, China). Metaphase chromosomes were GTG-banded using standard procedures.

#### Multiplex fluorescence in situ hybridization (M-FISH) analysis

M-FISH was performed on the metaphase spreads using Spectra Vysion WCP probe (Vysis, Inc., Downers Grove, IL, USA) according to manufacturer’s procedures. Images were captured with Olympus BX51 microscope (Olympus, Tokyo, Japan) and analyzed with the Cytovision 3.0 (Applied Imaging, Sunderland, UK) image analyses software.

#### OaCGH analysis

In order to investigate the extent of duplication on molecular level, analysis of using a genomic-wide high density oligo array (OaCGH244 K) was conducted according to Agilent manufacturer’s procedures and statistical algorithms (www.agilent.com.chem/gocgh) (9).

**Figure 3:**
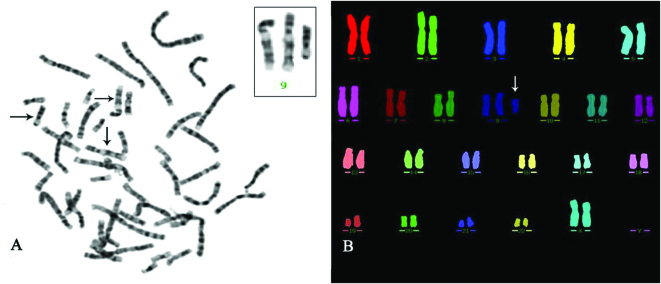
Chromosome analysis. (A) An extra der (9) (9pter→9q21) (arrowhead) by high-resolution G-banding (400-band level). (B) The extra chromosome from chromosome 9 (arrowhead) was confirmed by multiplex fluorescence *in situ* hybridization.

**Figure 4:**
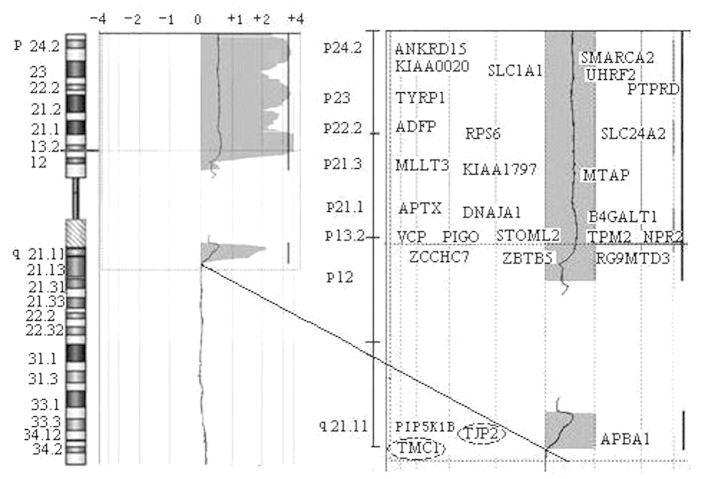
A 69.5 Mb duplication segment at genomic position 273,048-72,521,148 bp in the 9pter-q21.12 region was confirmed (Left). The duplicate region spanned 148 annotated genes in which 28 genes are expressed in the cochlea (Right). Dashed circles highlight the location of TMC1 and TJP2 on the map.

Chromosomal analysis. showed a female non-mosaic karyotype with an extra chromosome in all metaphases analyzed (Fig. 3A). M-FISH analysis using the Spectra Vysion WCP Probe (Vysis, Downers Grove, IL, USA) confirmed the extra chromosome from chromosome 9 (Fig. 3B). A 69.5 Mb duplication segment at genomic position 273,048 bp →72,521,148 bp in the 9pter→q21.12 region was confirmed (Fig. 4). The final karyotype was interpreted to be 47, XX, +mar.ish der (9) (wcp9+). arr cgh 9pterq21.12 (DOCK8→LOC138225)×3. The duplicate region spanned 148 annotated genes in which 28 genes are expressed in the cochlea (Fig. 4). Chromosome analysis of the parents showed normal karyotype, indicating a *de novo* extra chromosome.

## Discussion

To date, 65 genes for non-syndromic hearing loss have been identified (http://hereditaryhearingloss.org/) (10). However, to the best of our knowledge, hearing loss with isolated partial trisomy 9 (9pter→q21.12) has not been previously reported. The functions of the 28 genes identified in the chromosomal analysis, which are expressed in the cochlea, are mostly unknown. Reviewing the literature, cases of two males with partial trisomy 9, including duplication of 9per→q21 was reported by Morrissette *et al* (11) and Centerwall *et al* (12), respectively; however, the patients succumbed to the disease at four weeks following birth and thus it was uncertain whether or not hearing loss occurred. Comparing our case with other cases in the literature (2–8,13–18) it was found that the patients without hearing loss have overlapping regions of 9pter→9q13 or 9q22-9q32. On the basis of these data, it was hypothesized that 9q13-q21 may be a critical region for hearing. Recently, mutations of two genes in the region of 9q13-9q21.1 were confirmed to be responsible for deafness. For example, transmembrane channel-like gene 1 (TMC1, MIM 606706, GenBank ID NT_023935 position 4301249-4615799), mutations are identified by Kurima *et al* (19) as a cause of autosomal dominant (#MIM 606705) and autosomal recessive non-syndromic hearing loss (#MIM 600974). The association between mutations in the gene with hearing loss were further confirmed in other studies (20–23). Between 2002 and 2008, a total of 2 dominant and 18 recessive TMC1 mutations were reported as the cause of hearing loss in 34 families (24). Additionally, Hilgert *et al* (24) found the other six families with non-syndromic hearing loss were associated with mutations in DFNA36 and DFNB7/11, rather than mutations in TMC1, which implied at least one additional deafness-causing gene at loci DFNA36 and DFNB7/11. Another candidate gene, tight junction protein 2 (TJP2, MIM 607709), was considered a good candidate due to its function as a tight junction protein and its expression in the cochlea. Hilgert *et al* (24) reported a Guatemalan family with autosomal dominant nonsyndromic hearing loss. In exon 19 of the gene, a novel sequence variant, The mutation, c.2971A>T, was identified in the girl with the hearing loss phenotype, and this lead to an amino acid change from proline to valine at codon 924 (P924V). This aspartic acid residue is a member of a conserved acidic domain of the protein. The mutation was predicted to cause decreased stability by bioinformatic analysis. However, our hypothesis remains to be proven.

In addition to the typical clinical features of partial trisomy 9, the present case presented a group of distinctive phenotypes: The left side of the body was slightly smaller than the right one; left hearing loss was more severe than right; ptosis and strabismus of the left eye, all of which were not previously associated with partial trisomy 9. Body asymmetry is a complex developmental malformation and has already been described in syndromes, such as Beckwith-Wiedemann Syndrome (MIM 147470), Silver-Russell Syndrome (MIM 180860), Proteus syndrome (MIM 176920) and Klippel-Trenaunay-Weber syndrome (MIM 149000). Reviewing the literature, only one case of mosaic tetrasomy 9p with this anomaly was found (25). Considering the malformations are rare, it is uncertain whether the distinctive features were associated with partial trisomy 9 or not. However, the unusual clinical features with a detailed molecular karyotyping may provide information on this phenotype and expand existing knowledge.

In conclusion, the patient carrying a segmental duplication of 9pter-q21.12 exhibits distinctive phenotypes, such as sensorineural hearing loss. Although the molecular mechanism underlying the hearing loss is not clear, it was proposed that the region of 9q13→9q21 may be critical for hearing.
